# Physiological Mechanisms Underlying Chemical Fertilizer Reduction: Multiyear Field Evaluation of Microbial Biofertilizers in ‘Gala’ Apple Trees

**DOI:** 10.3390/plants15020244

**Published:** 2026-01-13

**Authors:** Susana Ferreira, Marta Gonçalves, Margarida Rodrigues, Francisco Martinho, Miguel Leão de Sousa

**Affiliations:** 1 National Institute for Agrarian and Veterinary Research (INIAV), Public Institute, Estrada de Leiria, 2460-059 Alcobaça, Portugal; marta.goncalves@iniav.pt (M.G.); margarida.rodrigues@iniav.pt (M.R.); francisco.martinho@iniav.pt (F.M.); 2 GREEN-IT—Bioresources for Sustainability, 2780-157 Oeiras, Portugal

**Keywords:** chlorophyll fluorescence, gas exchange, leaf morphology, *Malus domestica*, Mediterranean orchards, photosynthetic efficiency, spectral indices, stress tolerance

## Abstract

This study is Part II of a five-year (2018–2022) field trial in western Portugal evaluating the effects of three microbial biofertilizers—Mycoshell^®^ (*Glomus* spp. + humic/fulvic acids), Kiplant iNmass^®^ (*Azospirillum brasilense*, *Bacillus megaterium*, *Saccharomyces* cerevisiae), and Kiplant All-Grip^®^ (*Bacillus megaterium*, *Pseudomonas* spp.)—applied at different dosages alongside two mineral fertilizer regimes, T100 (full dose) and T70 (70% of T100, alone or combined with biofertilizers), on the physiological performance of ‘Gala Redlum’ apple trees. Part I had shown that Myc4 (Mycoshell^®^, 4 tablets/tree), iNM6, and iNM12 (Kiplant iNmass^®^, 6 and L ha^−1^, respectively) consistently enhanced fruit growth, yield, and selected quality traits. While Part I showed clear agronomic gains, Part II demonstrates that these improvements occurred without significant alterations in seasonal photosynthetic performance, canopy reflectance, or chlorophyll fluorescence parameters over five years, highlighting the contrast between observed yield improvements and physiological stability. Seasonal monitoring of physiological traits—including specific leaf area (SLA), chlorophyll content index (CCI), gas exchange (*A_n_*, *g_s_*, *E*, *C_i_*), spectral indices (NDVI, OSAVI, SIPI, GM2), and chlorophyll fluorescence (OJIP). It is clear that physiological values remained largely stable across biofertilizer treatments and years. Importantly, this stability was maintained even under a 30% reduction in mineral fertilizer (T70), indicating that specific microbial biofertilizers can sustain physiological resilience under reduced nutrient inputs, thereby providing a physiological basis for the yield-enhancing effects observed and supporting their integration into fertilizer reduction strategies in Mediterranean orchards.

## 1. Introduction

Efforts to reduce mineral fertilizer inputs and integrate biofertilizers into perennial cropping systems have intensified [1]. This need is pressing in Mediterranean regions, where climate variability and recurrent droughts require resilient orchard management [2,3]. Biofertilizers—containing microorganisms such as arbuscular mycorrhizal fungi [4], phosphate-solubilizing bacteria [5], and diazotrophic bacteria [6]—can enhance nutrient acquisition, modulate phytohormonal pathways, and increase tolerance to abiotic stress [7,8,9,10]. These physiological improvements are critical for sustaining canopy vigor, productivity, and fruit quality under reduced fertilization regimes [11,12].

Multiyear field studies in ‘Gala’ apple orchards indicate that combining biofertilizers with reduced mineral fertilization maintains or improves soil fertility, leaf nutrient status, and agronomic performance. However, the underlying physiological mechanisms remain poorly understood [13,14,15]. Most existing studies focus either on yield responses or on isolated physiological parameters, often under short-term or controlled conditions. In contrast, multi-year, in-field assessments integrating multiple biofertilizer formulations, a quantified reduction in mineral fertilization, and a comprehensive physiological monitoring framework across key phenological stages remain scarce.

Major knowledge gaps include the lack of standardization in biofertilizer production, limited understanding of the microbiota in each formulation, and insufficient capacity to fully replace chemical fertilizers [16,17,18]. These limitations hinder the development of robust, physiology-based recommendations for orchard management. Addressing these limitations is crucial to validate biofertilizers not only as nutrient supplements but also as agents of physiological optimization under field conditions [19].

Recent advances in non-invasive physiological monitoring provide an opportunity to address these knowledge gaps under field conditions [20,21,22,23,24,25,26,27]. By integrating chlorophyll fluorescence (OJIP parameters), canopy spectral reflectance indices (e.g., NDVI, OSAVI, PRI), and gas exchange measurements, it is possible to assess photosynthetic performance, canopy function, and carbon–water relations in a comprehensive and non-destructive manner across key phenological stages. However, such an integrated physiological toolkit has rarely been applied in multi-year field trials evaluating microbial biofertilizers under reduced mineral fertilization in perennial fruit crops.

This study presents physiological results from the second part of a five-year field experiment in western Portugal, focusing on ‘Gala Redlum’ apple trees subjected to biofertilizer treatments under reduced mineral fertilization [13]. Building upon agronomic and soil-based findings previously reported [28], we explore here the physiological dimension of biofertilizer application. Specifically, we assess leaf morphology (specific leaf area (SLA)), chlorophyll content (CCI), gas exchange parameters, spectral indices, and chlorophyll fluorescence across multiple treatments and seasons [29].

We hypothesize that biofertilizers enhance photosynthetic efficiency, stomatal conductance, and pigment-related traits, with such enhancements likely most pronounced under summer conditions [30]. By linking microbial treatments to physiological outcomes, this work aims to elucidate the functional mechanisms that support orchard resilience and productivity under Mediterranean conditions [31]. These insights contribute to the development of integrated fertilization strategies aligned with the European Union Farm-to-Fork objectives and the long-term sustainability of fruit production systems [32].

## 2. Results

Measurements were conducted across multiple growing seasons to evaluate the physiological responses of ‘Gala’ apple trees to different biofertilizer treatments. Three key phenological stages were considered: spring (active leaf expansion and early vegetative growth), summer (full canopy development and peak photosynthetic activity), and a limited autumn/post-harvest (senescence) stage in 2018, in which only chlorophyll content was measured to provide additional context. All other physiological parameters were measured from 2019 onwards. Not all seasons were assessed in every year, and some seasons included multiple sampling dates; specific measurement dates, instruments, and replicate numbers are provided in the Materials and Methods Section. This temporal framework enables a robust, season-resolved evaluation of treatment effects on leaf traits, pigment content, and overall physiological performance.

### 2.1. Specific Leaf Area (SLA)

**Figure 1:**
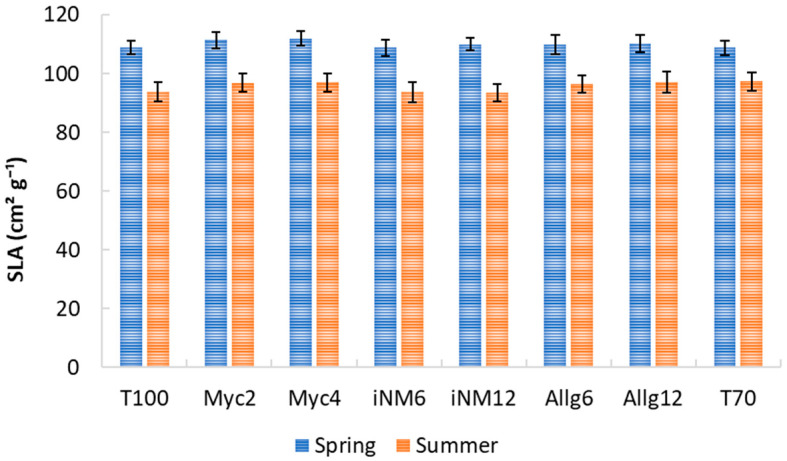
Average SLA of ‘Gala’ apple leaves under different treatments in spring and summer (2019–2022). Error bars represent the standard error of the mean (SEM).

Specific Leaf Area varied significantly with year and season, but biofertilizer treatments had no consistent main effect. SLA was assessed as the four-year mean (2019–2022) for the spring and summer seasons (Figure 1). Overall, SLA was higher in spring across all treatments, reflecting the presence of younger, thinner leaves, and decreased in summer, when leaves were more mature. In spring, the highest mean SLA values were observed in Myc4 (111.94 cm^2^ g^−1^) and Myc2 (111.41 cm^2^ g^−1^), while the lowest values occurred in T70 (108.80 cm^2^ g^−1^) and iNM6 (108.77 cm^2^ g^−1^). During summer, mean SLA values across treatments ranged from 93.54 cm^2^ g^−1^ (iNM12) to 97.40 cm^2^ g^−1^ (T70).

Because spring and summer leaves differ markedly in age and morphology, data were analyzed separately for each season. Two-way ANOVA (Table A1) revealed a strong year effect in both seasons, whereas treatment effects were negligible. Significant Treatment × Year interactions reflected only minor variations among treatments across years. Complementary analyses using Welch’s ANOVA and non-parametric tests confirmed that temporal (year-to-year) variation overwhelmingly explains SLA differences, while fertilization treatments had no consistent effect.

### 2.2. Chlorophyll Content Index (CCI)

**Figure 2:**
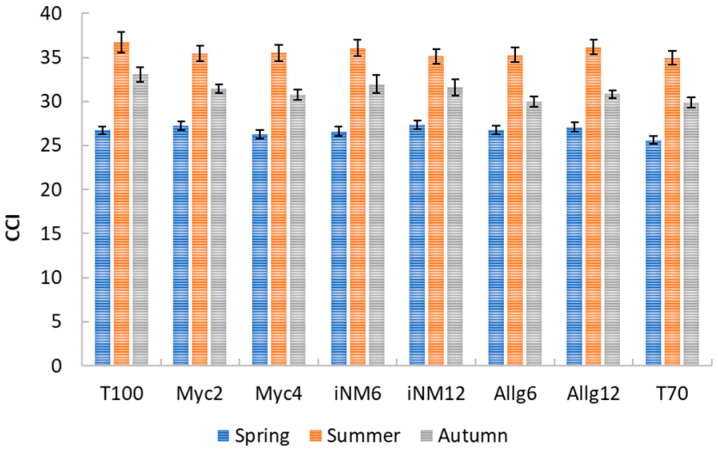
Average CCI of ‘Gala’ apple leaves under different treatments in spring and summer (2019–2022). Error bars represent the standard error of the mean (SEM).

Chlorophyll Content Index was primarily influenced by interannual and seasonal variability, while treatment effects were minor and inconsistent. Descriptive statistics for the CCI across treatments and seasons are presented in Figure 2. In spring, mean CCI values ranged from 25.62 ± 0.46 (T70) to 27.33 ± 0.47 (iNM12; values reported as mean ± SEM). During summer, values increased to 34.94 ± 0.80 (T70)–36.69 ± 1.18 (T100), reflecting higher chlorophyll content during peak growth. In autumn, values were intermediate, ranging from 29.90 ± 0.58 (T70) to 33.09 ± 0.83 (T100). No significant differences were observed in spring or summer.

Because spring and summer leaves differ in age and morphology, seasonal data were analyzed separately (Table 1). In spring, CCI varied significantly among years (highest in 2019, progressively lower in 2020 and 2022), with no treatment effect. During summer, interannual variability largely drove CCI patterns (Year effect: F_3,416_ = 114.51, *p* < 0.001, η^2^p = 0.452), whereas treatment effects remained minor (F_7,416_ = 0.72, *p* = 0.655, η^2^p = 0.012). Post hoc analyses indicated that 2022 had higher CCI values across most treatments, while differences among treatments within each year were negligible.

In autumn 2018, leaf CCI showed small but significant differences among treatments (ANOVA, F_7,152_ = 2.306, *p* = 0.029, η^2^p = 0.096), with T100 slightly higher than T70 (mean difference = 3.193, *p* = 0.032). Levene’s test confirmed homogeneity of variances (F_7,152_ = 1.244, *p* = 0.282). Overall, these results suggest that CCI was primarily shaped by seasonal and interannual conditions, with treatment effects limited and mostly year-specific. A significant Year × Treatment interaction (F_21,416_ = 2.365, *p* < 0.001, η^2^p = 0.107) indicates minor treatment-specific annual responses.

### 2.3. Gas Exchange Parameters

**Figure 3:**
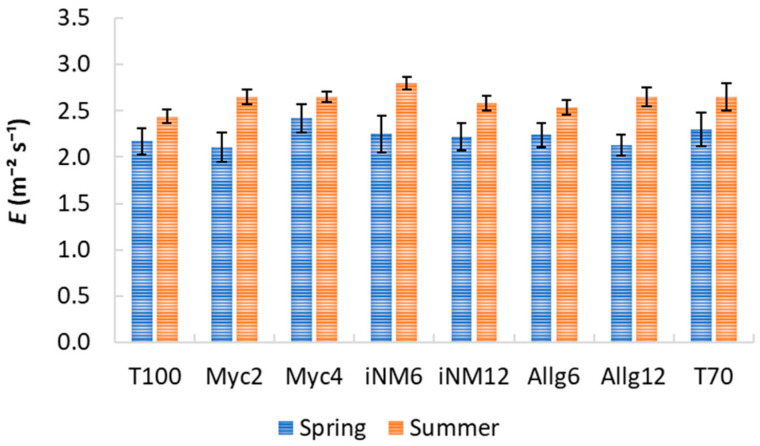
*E* (mmol m^−2^ s^−1^) for treatments across four growing seasons (2019–2022). Error bars represent SEM.

**Figure 4:**
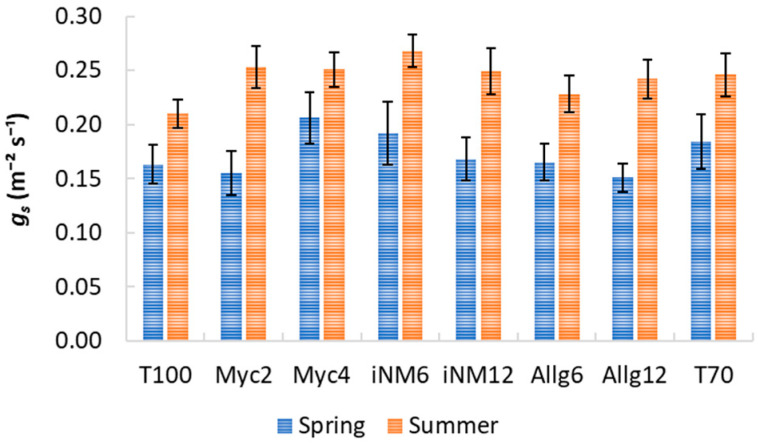
*gs* (mol m^−2^ s^−1^) for treatments across four growing seasons (2019–2022). Error bars represent SEM.

**Figure 5:**
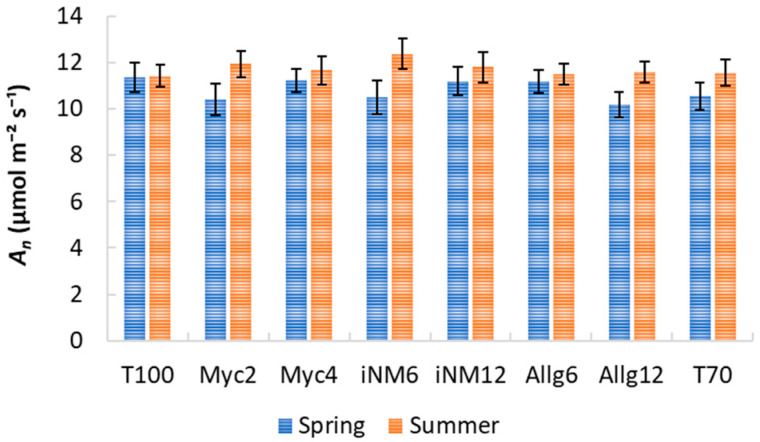
*A_n_* (µmol m^−2^ s^−1^) for treatments across four growing seasons (2019–2022). Error bars represent SEM.

Gas exchange parameters exhibited clear interannual trends, but biofertilizer treatments had a negligible impact. Descriptive statistics for intercellular CO_2_ concentration (*C_i_*), water-use efficiency (WUE), transpiration (*E*), stomatal conductance (*g_s_*), and net photosynthetic rate (*A_n_*) across treatments and years are shown in Table 2 and Table 3 and Figure 3, Figure 4 and Figure 5. Mean *C_i_* ranged from 232.42 µmol mol^−1^ (Allg6, 2022) to 282.33 µmol mol^−1^ (Myc4, 2021), with minor treatment differences and clear interannual variability. WUE varied from 3.71 µmol CO_2_ mmol^−1^ H_2_O (T70, 2021) to 5.37 µmol CO_2_ mmol^−1^ H_2_O (Allg6, 2022), showing higher values in 2020 and 2022. *E*, *g_s_*, and *A_n_* all increased from spring to summer, with Myc4, iNM6, and iNM12 generally exhibiting the highest *g_s_* and *A_n_*, while T100 and Allg6 showed smaller seasonal increases.

ANOVA confirmed year effects for all variables, while treatment and Treatment × Year interactions were non-significant (Table A2). Post hoc tests highlighted interannual trends (e.g., WUE 2019 < 2022, 2020 > 2021).

PCA was performed to explore multivariate relationships among gas exchange parameters (Table 4). KMO (0.51) and Bartlett’s test (χ^2^_10_ = 1851.27, *p* < 0.001) confirmed the suitability. Two principal components explained 95.7% of the variance: RC1 (48.8%) associated with photosynthetic activity (*A_n_*, *g_s_*, *E*), and RC2 (46.9%) representing the WUE–*C_i_* trade-off. Myc4, iNM6, and iNM12 treatments had higher RC1 scores (enhanced photosynthesis and stomatal conductance), whereas T100 and iNM12 scored higher on RC2 (greater WUE, lower *C_i_*). ANOVA on PCA scores revealed no significant differences among treatments (PC1: F_7,248_ = 0.45, *p* = 0.87; PC2: F_7,248_ = 0.97, *p* = 0.46), confirming physiological stability across biofertilizer applications over four years.

### 2.4. Leaf Reflectance Indices

Leaf reflectance indices showed moderate interannual variation, reflecting seasonal canopy development, with minimal and inconsistent treatment effects. Although individual spectral indices did not differ significantly among treatments in univariate ANOVA (Table A3), PCA revealed coherent multivariate patterns reflecting canopy physiology, pigment composition, and stress responses. Seasonal PCA was conducted separately for spring and summer to account for structural and physiological differences between developing and mature foliage.

In spring, PCA (KMO = 0.84; Bartlett’s χ^2^(300) = 2575.6, *p* < 0.001) retained two principal components (RC1–RC2) explaining 87.1% of the variance. RC1 captured canopy vigor and structural development (e.g., NDVI, GM2, TCARI), while RC2 represented pigment- and stress-related variability (e.g., PRI, ARI indices). Some rotated loadings exceeded 1 due to the oblique Promax rotation, reflecting component correlations rather than errors (Table 5).

For summer, PCA (KMO = 0.67; Bartlett’s χ^2^(300) = 2520.1, *p* < 0.001) retained three components explaining 92.4% of the variance. RC1 remained associated with canopy vigor and greenness, RC2 reflected pigment-related variation, and RC3 captured chlorophyll absorption and stress response (Table 6). Again, oblique rotations produced some loadings > 1, which are mathematically valid.

ANOVA on PCA scores confirmed no significant treatment effects for any component in either season (spring PC1–PC2, *p* > 0.99; summer PC1–PC3, *p* > 0.95). Consequently, ANOVA on the PCA scores revealed no significant treatment effects for any component in either season, confirming that canopy spectral properties were not altered by biofertilizer application. Thus, while biofertilizer treatments did not alter individual spectral indices or overall PCA scores, multivariate analyses captured physiological gradients influenced by canopy development and seasonal dynamics.

**Figure 6:**
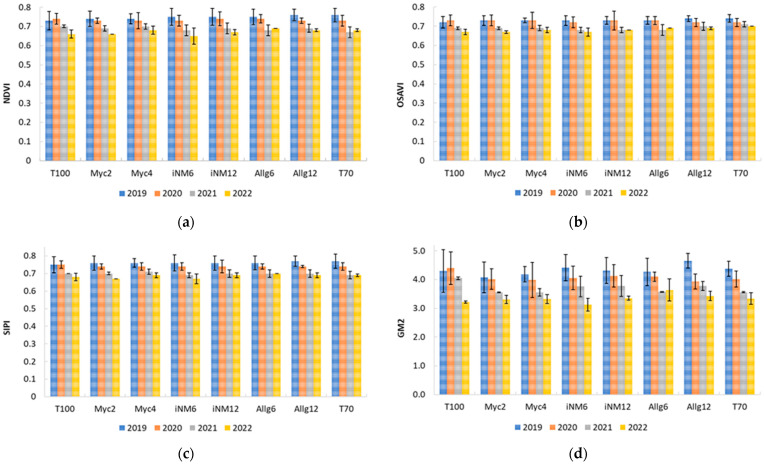
Annual evolution of vegetation indices in the different treatments from 2019 to 2022: (**a**) NDVI, (**b**) OSAVI, (**c**) SIPI, (**d**) GM2.

From the PCA, four representative indices were selected for temporal analysis (2019–2022): NDVI and OSAVI (vegetation vigor), SIPI (carotenoid-to-chlorophyll ratio), and GM2, which is sensitive to chlorophyll content and also captures aspects of canopy structure. Across four years, indices showed slight interannual variation and a gradual reduction in canopy vigor and pigment-related traits. NDVI and OSAVI decreased from 0.73 to 0.76 (2019–2020) to 0.65–0.67 (2021–2022) in T100 and iNM6, while SIPI declined from 0.75 to 0.77 to 0.67–0.70. GM2 exhibited higher sensitivity, declining from 4.7 (Allg12, 2019) to 3.1 (iNM6, 2022) (Figure 6).

These results suggest a moderate interannual decline in canopy vigor and pigment content, with GM2 being particularly sensitive to structural changes. Overall, biofertilizer treatments had minimal effect, while seasonal and environmental factors largely drove reflectance patterns, aligning with trends observed in physiological and leaf functional traits.

### 2.5. Chlorophyll Fluorescence Parameters

Chlorophyll fluorescence parameters were strongly shaped by year-to-year and seasonal variation, whereas biofertilizer treatments had only minor and unstable effects. Descriptive statistics for chlorophyll fluorescence parameters measured in spring and summer are presented in Table 7 and Table 8, respectively. These parameters include the maximum quantum yield of photosystem II (*Fv*/*Fm*), the quantum yield of electron transport (*φ_Eo_*), the quantum yield of energy dissipation (*φ_Do_*), the performance index on absorption basis (PI _ABS_), and related energy flux parameters per reaction center: absorption flux (ABS/RC), trapped energy flux (TRo/RC), electron transport flux (ETo/RC), and dissipated energy flux (DIo/RC).

In spring (Table 7), *Fv*/*Fm* values ranged from 0.79 to 0.81 across treatments, with T100 and Myc4 showing slightly lower values (0.79 ± 0.01) compared to Myc2 and iNM12 (0.81 ± 0.01). The performance index (PI _ABS_) followed a similar trend, with the highest values in iNM12 (5.03 ± 0.37) and Myc2 (5.25 ± 0.42), while T100 exhibited lower performance (4.59 ± 0.40). Other chlorophyll fluorescence parameters varied minimally among treatments, with iNM6 and iNM12 generally showing slightly higher electron transport (TRo/RC and ETo/RC) and slightly lower energy dissipation (DIo/RC) compared to Allg and T70 treatments, suggesting more efficient photochemical activity.

**Table 7:** Descriptive statistics (Mean ± SEM) of chlorophyll fluorescence parameters for different treatments in spring.

Treatment	*Fv*/*Fm*	*φ_Eo_*	*φ_Do_*	PI _ABS_	ABS/RC	TRo/RC	ETo/RC	DIo/RC
T100	0.79 ± 0.01	0.49 ± 0.01	0.21 ± 0.01	4.59 ± 0.40	1.47 ± 0.03	1.16 ± 0.02	0.71 ± 0.02	0.32 ± 0.01
Myc2	0.81 ± 0.01	0.50 ± 0.01	0.19 ± 0.01	5.25 ± 0.42	1.42 ± 0.02	1.15 ± 0.02	0.71 ± 0.02	0.27 ± 0.01
Myc4	0.79 ± 0.01	0.48 ± 0.01	0.20 ± 0.01	4.39 ± 0.36	1.53 ± 0.05	1.22 ± 0.03	0.73 ± 0.02	0.31 ± 0.01
iNM6	0.80 ± 0.01	0.48 ± 0.01	0.20 ± 0.01	4.53 ± 0.33	1.47 ± 0.04	1.18 ± 0.03	0.71 ± 0.02	0.29 ± 0.01
iNM12	0.80 ± 0.01	0.49 ± 0.01	0.19 ± 0.01	5.03 ± 0.37	1.41 ± 0.05	1.13 ± 0.03	0.68 ± 0.02	0.28 ± 0.01
Allg6	0.79 ± 0.01	0.47 ± 0.01	0.21 ± 0.01	4.05 ± 0.25	1.47 ± 0.04	1.17 ± 0.03	0.69 ± 0.02	0.31 ± 0.01
Allg12	0.80 ± 0.01	0.48 ± 0.01	0.20 ± 0.01	4.78 ± 0.46	1.42 ± 0.04	1.13 ± 0.03	0.68 ± 0.02	0.29 ± 0.01
T70	0.80 ± 0.01	0.48 ± 0.01	0.20 ± 0.01	4.56 ± 0.46	1.43 ± 0.04	1.14 ± 0.02	0.67 ± 0.02	0.29 ± 0.01

In summer (Table 8), *Fv*/*Fm* values were slightly higher and less variable, ranging from 0.80 to 0.82. T100 and iNM12 displayed the highest PI _ABS_ values (5.77 ± 0.67 and 5.93 ± 0.40, respectively), whereas Myc4 and T70 were slightly lower (5.14 ± 0.42 and 5.01 ± 0.42). Energy fluxes per reaction center exhibited similar patterns to spring, with iNM12 and Myc2 tending to maintain higher TRo/RC and lower DIo/RC values, indicating efficient use of absorbed energy. Overall, the *Fv*/*Fm* ratio remained relatively stable across treatments and seasons, reflecting maintained photosynthetic efficiency.

**Table 8:** Descriptive statistics (Mean ± SEM) of chlorophyll fluorescence parameters for different treatments in summer.

Treatment	*Fv*/*Fm*	*φ_Eo_*	*φ_Do_*	PI _ABS_	ABS/RC	TRo/RC	ETo/RC	DIo/RC
T100	0.81 ± 0.01	0.51 ± 0.01	0.18 ± 0.01	5.77 ± 0.67	1.30 ± 0.03	1.10 ± 0.03	0.63 ± 0.01	0.23 ± 0.01
Myc2	0.81 ± 0.01	0.50 ± 0.01	0.19 ± 0.01	5.49 ± 0.43	1.49 ± 0.04	1.37 ± 0.03	0.69 ± 0.01	0.26 ± 0.01
Myc4	0.81 ± 0.01	0.49 ± 0.01	0.19 ± 0.01	5.14 ± 0.42	1.37 ± 0.03	1.37 ± 0.03	0.67 ± 0.01	0.26 ± 0.01
iNM6	0.80 ± 0.01	0.48 ± 0.01	0.20 ± 0.01	4.92 ± 0.49	1.37 ± 0.03	1.10 ± 0.03	0.66 ± 0.01	0.27 ± 0.01
iNM12	0.81 ± 0.01	0.51 ± 0.01	0.19 ± 0.01	5.93 ± 0.40	1.33 ± 0.03	1.08 ± 0.03	0.68 ± 0.01	0.25 ± 0.01
Allg6	0.81 ± 0.01	0.49 ± 0.01	0.19 ± 0.01	5.46 ± 0.61	1.40 ± 0.03	1.12 ± 0.03	0.68 ± 0.01	0.27 ± 0.01
Allg12	0.82 ± 0.01	0.48 ± 0.01	0.18 ± 0.01	5.18 ± 0.64	1.37 ± 0.04	1.12 ± 0.03	0.66 ± 0.01	0.25 ± 0.01
T70	0.80 ± 0.01	0.49 ± 0.01	0.20 ± 0.01	5.01 ± 0.42	1.37 ± 0.03	1.37 ± 0.03	0.67 ± 0.01	0.26 ± 0.01

The overall patterns of year and interaction effects on *Fv*/*Fm*, *φ_Eo_*, *φ_Do_*, PI _ABS_, ABS/RC, TRo/RC, ETo/RC, and DIo/RC are summarized in Table A4.

Interannual effects were more pronounced than treatment effects. For spring, *Fv*/*Fm* differed significantly among years (F_3,184_ = 14.85, *p* < 0.001, η^2^_p_ = 0.20), with means of 0.80 ± 0.02 (2019), 0.82 ± 0.02 (2020), 0.79 ± 0.03 (2021), and 0.78 ± 0.03 (2022). Significant interannual variation was also observed for *φ_Do_* (F = 11.93, *p* < 0.001, η^2^_p_ = 0.14), PI _ABS_ (F = 5.36, *p* = 0.006, η^2^_p_ = 0.07), ABS/RC (F = 22.48, *p* < 0.001, η^2^_p_ = 0.24), TRo/RC (F = 24.54, *p* < 0.001, η^2^_p_ = 0.25), ETo/RC (F = 16.51, *p* < 0.001, η^2^_p_ = 0.17), and DIo/RC (F = 16.69, *p* < 0.001, η^2^_p_ = 0.19), highlighting clear interannual differences in photosynthetic performance. *φ_Eo_* did not vary significantly among years in spring.

In summer, significant interannual variability was observed for *Fv*/*Fm* (F_3,165_ = 26.37, *p* < 0.001, η^2^_p_ = 0.25), *φ_Eo_* (F = 10.55, *p* < 0.001, η^2^_p_ = 0.12), *φ_Do_* (F = 33.02, *p* < 0.001, η^2^_p_ = 0.29), PI _ABS_ (F = 15.76, *p* < 0.001, η^2^_p_ = 0.16), ABS/RC (F = 7.91, *p* < 0.001, η^2^_p_ = 0.09), TRo/RC and DIo/RC (F = 18.25, *p* < 0.001, η^2^_p_ = 0.18), whereas ETo/RC remained relatively stable. These results indicate that year-to-year variation, rather than biofertilizer treatment, was the main driver of differences in photochemical parameters.

**Figure 7:**
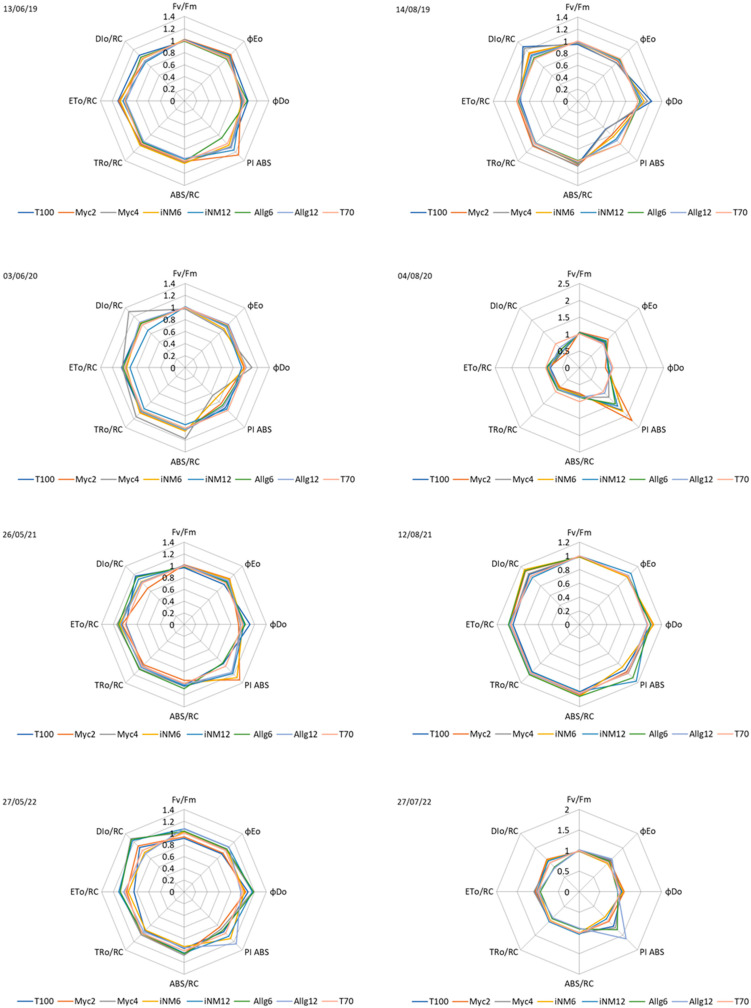
Radar plot of normalized photochemical parameters (*Fv*/*Fm*, *φ_Eo_*, *φ_Do_*, PI _ABS_, ABS/RC, TRo/RC, ETo/RC, and DIo/RC). Data are normalized to T70 for each parameter. Each axis represents a parameter, and each line corresponds to a treatment. The measurement date is indicated in the top-left corner of each plot. Seasons are defined as spring (before 22 June) and summer (after 22 June).

Seasonal trends were visualized using radar graphs for each year (Figure 7). Across 2019–2022, spring measurements, normalized to the control treatment (T70), showed higher PSII performance (*Fv*/*Fm* ≈ 0.99–1.02, *φ_Eo_* > 1.05, PI _ABS_ ≈ 1.10–1.30), while summer displayed physiological declines, with lower *φ_Eo_* and PI _ABS_ (often <1.00) and increased DIo/RC (>1.15 under midsummer conditions).

The magnitude of treatment effects varied among years. In 2020, spring PI _ABS_ was lower (0.59–0.84), while summer showed the largest treatment differences (PI _ABS_ up to 2.21 in Myc2, *φ_Eo_* up to 1.20). In 2022, summer variability was also high (PI _ABS_ = 1.12–1.61, with Allg12 standing out). In contrast, 2019 and 2021 exhibited smaller inter-treatment differences, especially in spring, with summer clustering around the control.

Overall, treatment effects were inconsistent, and the year × treatment interaction was non-significant. These results indicate that seasonality and interannual climatic variability were the main drivers of chlorophyll fluorescence patterns, with minor and unstable biofertilizer effects.

## 3. Discussion

### 3.1. SLA and Temporal Variation

SLA exhibited clear seasonal and interannual variability, with higher values in spring compared to summer and a general upward trend from 2019 to 2022. Spring leaves, being younger and thinner, naturally showed higher SLA, whereas summer leaves were more mature and structurally denser, reflecting ontogenetic changes [33]. Statistical analyses revealed a very strong Year effect in both seasons, with no consistent main effect of treatment, although Treatment × Year interactions suggested minor modulation of treatment responses by annual conditions.

Considering local meteorological data, these patterns are plausible. For instance, spring mean temperatures were slightly higher in 2022 compared to 2019, while spring rainfall was lower in 2021–2022, yet SLA increased, suggesting that factors beyond simple water availability, such as the timing and rate of leaf expansion, likely influenced leaf structure [34]. Summers in 2020–2022 were characterized by higher evaporative demand (ET_o_ ~4.8 mm d^−1^) and reduced rainfall, conditions that typically promote leaf thickening and reduced SLA [35]; however, SLA decreased in summer but remained higher than expected, suggesting potential buffering by orchard management, carry-over effects from spring, or dominance of phenology-driven structural traits.

Overall, the combined evidence from ANOVA, Welch, and non-parametric tests indicates that SLA was primarily shaped by interannual climatic variation and leaf ontogeny rather than the applied treatments, with any treatment effects being subtle and year-specific. These results underline the importance of considering seasonal timing, heteroscedasticity, and temporal climatic patterns when interpreting SLA responses, while acknowledging that causal links between SLA and meteorological variables remain speculative without finer-scale soil moisture, irrigation, and phenological data [36].

### 3.2. Discussion of CCI in Relation to Monthly Climate Variables

The CCI of ‘Gala’ apple leaves exhibited clear seasonal and interannual variability across the four-year period. In spring, CCI values were moderate and primarily influenced by interannual conditions rather than soil management treatments, with 2019 showing the highest and 2022 the lowest values. This trend mirrors climatic patterns, where spring temperatures (T_mean_ 12.2–17.8 °C) and rainfall fluctuations likely affected leaf chlorophyll synthesis and nitrogen availability [37]. Negligible treatment effects suggest young leaves are more sensitive to environmental cues than to soil management, consistent with findings in other perennial fruit systems [38].

During summer, CCI increased compared with spring, reflecting chlorophyll accumulation in fully expanded leaves under peak photosynthetic demand. Interannual differences were pronounced in 2022, when higher temperatures (T_mean_ 18.8–21.8 °C), moderate rainfall, and high radiation (RS 18.4–27.1 MJ m^−2^ d^−1^) likely enhanced chlorophyll across all treatments [39]. Despite detectable Treatment × Year interactions, the main effect of soil management was minimal, indicating climatic conditions overrode potential effects of mineral fertilization [40].

Autumn CCI values were intermediate and showed small but significant differences among treatments in 2018, with T100 slightly higher than T70, suggesting higher nutrient input or soil fertility may have delayed leaf senescence [41]. The significant Year × Treatment interaction indicates subtle treatment-specific responses depending on climate, but interannual variability remained dominant. Overall, CCI dynamics underscore the influence of seasonal development and year-specific meteorology on leaf chlorophyll content, highlighting the importance of accounting for climatic context when interpreting treatment effects [42].

### 3.3. Gas Exchange Responses to Biofertilizer Treatments

The analysis of gas exchange parameters (*C_i_*, WUE, *E*, *g_s_*, and *A_n_*) across four growing seasons indicated that interannual variability had a stronger influence than biofertilizer treatments, highlighting the primary role of environmental conditions in regulating apple tree physiology. Mean *C_i_* values ranged from low levels in 2022 (e.g., Allg6, T70) to maxima in 2021 (e.g., Myc4), likely reflecting the combined effects of spring temperature, radiation, and evapotranspiration demands [43].

Water-use efficiency followed a similar interannual pattern, with the highest values in 2020 and 2022 and the lowest in 2019 and 2021. Elevated WUE in 2022 may reflect a compensatory response to higher summer evaporative demand, leading to tighter stomatal regulation while maintaining photosynthesis [44]. In contrast, lower WUE in 2021 corresponds with moderate rainfall and radiation, suggesting that under favorable water conditions, carbon gain per unit water is naturally reduced. These trends indicate that seasonal water availability and atmospheric demand are the main constraints on WUE in apple trees rather than soil treatments.

Transpiration and stomatal conductance consistently increased from spring to summer across all treatments, reflecting typical seasonal responses to temperature, radiation, and vapor pressure deficit [45]. Spring *g_s_* and *E* declined from 2020 to 2022, with 2022 showing the lowest values, consistent with lower soil moisture and higher vapor pressure deficit [46]. These shifts explain the observed interannual differences in net photosynthesis, with higher *A_n_* under favorable spring conditions (e.g., 2020) and a summer decline in 2022, suggesting both stomatal and non-stomatal limitations under stress.

PCA results corroborated these observations, with two components explaining 95.7% of the variance. RC1, associated with *A_n_*, *g_s_*, and *E*, reflects overall photosynthetic performance, while RC2 captures the trade-off between *C_i_* and WUE, representing physiological strategies to optimize carbon gain relative to water use. Treatments such as Myc4, iNM6, and iNM12 scored higher on RC1, indicating enhanced photosynthesis and stomatal conductance, whereas T100 and iNM12 scored higher on RC2, reflecting more conservative water use. ANOVA on PCA scores confirmed no significant differences among treatments, demonstrating physiological stability across biofertilizer applications over four years.

Overall, these results indicate that ‘Gala’ apple trees maintained consistent gas exchange performance under the applied soil management strategies, with environmental variability—temperature, radiation, and rainfall distribution—being the primary driver of photosynthetic activity and water relations. These patterns align with previously observed trends in CCI and SLA, further emphasizing the overarching influence of climatic conditions on canopy physiology and functional leaf traits [47].

### 3.4. Leaf Reflectance Indices and Physiological Responses

The analysis of leaf reflectance indices over the four-year period revealed that individual spectral indices did not differ significantly among treatments in univariate ANOVA, suggesting that overall canopy reflectance traits were largely resilient to the type of biofertilizer applied. However, multivariate PCA identified coherent physiological gradients, capturing subtle variations in canopy structure, pigment composition, and stress responses that were not detectable through single-index analysis. Seasonal PCAs were particularly informative, highlighting the marked differences between developing spring foliage and mature summer canopies, which otherwise introduce confounding variability when pooled across seasons.

In spring, RC1 primarily reflected canopy vigor and structural development, while RC2 represented pigment- and stress-related variability. This separation aligns with the physiological distinction between young, actively expanding leaves and mature leaf cohorts later in the season, consistent with the seasonal dynamics observed in CCI and gas exchange parameters (*A_n_*, *g_s_*, *E*) [13]. In summer, three components captured canopy vigor (RC1), pigment-related variation (RC2), and chlorophyll absorption/stress response (RC3), confirming that mature canopies present more complex interactions between structural traits and photoprotective mechanisms [48]. The absence of significant differences in PCA-derived component scores among treatments indicates that, despite subtle gradients, biofertilizer application did not induce major alterations in leaf spectral properties.

Temporal analysis of selected indices—NDVI, OSAVI, SIPI, and GM2—highlighted interannual patterns consistent with environmental conditions. NDVI and OSAVI, indicators of overall vegetation vigor, remained relatively stable in 2019–2020 but declined in 2021–2022, reflecting the impact of interannual variability in meteorological conditions on canopy development [49]. SIPI, indicative of carotenoid-to-chlorophyll ratio and stress status, exhibited a similar temporal decline, suggesting subtle pigment adjustments in response to cumulative environmental factors [50]. GM2, reflecting canopy structural traits and chlorophyll content, displayed the most pronounced interannual variation, with the highest value recorded in Allg12 (2019) and the lowest in iNM6 (2022), indicating that canopy architecture may be more sensitive to environmental fluctuations than pigment-based indices [51].

The observed temporal trends align with interannual patterns of CCI and gas exchange parameters, reinforcing the notion that environmental conditions—particularly light availability, temperature, and water status—play a more decisive role than biofertilizer type in shaping canopy reflectance properties [52]. The slight decline in NDVI, OSAVI, and SIPI over the later years may be associated with cumulative effects of variable spring–summer conditions, including rainfall and temperature differences, which also affected photosynthetic performance and leaf water relations [53]. GM2’s responsiveness highlights its utility for detecting structural changes in canopy development that are not directly mirrored by pigment-related indices [54].

Overall, the multivariate PCA approach proved essential in revealing subtle physiological gradients, underscoring the importance of integrating seasonal and interannual perspectives when assessing treatment effects on complex canopy traits.

### 3.5. Seasonal Patterns in PSII Performance

Chlorophyll fluorescence measurements across four years (2019–2022) showed that photochemical efficiency, energy absorption, and dissipation traits were largely stable across biofertilizer treatments, with only subtle and inconsistent effects detected in spring (PC3). The maximum quantum yield of PSII (*Fv*/*Fm*) remained relatively constant (0.79–0.82), indicating preserved primary photochemistry and absence of chronic photoinhibition. Similarly, the performance index (PI _ABS_) and energy flux parameters (ABS/RC, TRo/RC, ETo/RC, DIo/RC) varied little, suggesting the overall balance between energy capture, photochemical utilization, and dissipation was maintained independently of biofertilizer type [55].

Seasonal trends were consistent across years. In spring, measurements reflected the physiological advantage of young, actively developing leaves, which typically present high photosynthetic efficiency and optimal PSII functioning [56]. In summer, despite higher irradiance and temperature, our results did not indicate a decline in PSII performance. Instead, treatments often showed stable or even higher *Fv*/*Fm* and PI _ABS_ values. This pattern suggests acclimation of mature leaves through photoprotective adjustment, as indicated by the increase in DIo/RC, which reflects enhanced energy dissipation under stress conditions [57], rather than loss of photochemical capacity.

Interannual variability was a major driver of chlorophyll fluorescence dynamics. Significant differences among years were observed for *Fv*/*Fm*, *φ_Do_*, PI _ABS_, and energy fluxes, reflecting climatic influences such as temperature, radiation, and water availability [58]. For example, spring 2020 showed lower PI _ABS_, while summer 2020 and 2022 exhibited transient increases in PI _ABS_ and *φ_Eo_*, demonstrating the plasticity of photochemical performance under yearly environmental fluctuations [59].

PCA analyses provided a multivariate perspective. In spring, three components explained 95.2% of the variance: RC1 captured the balance between photochemical efficiency and energy dissipation, RC2 reflected variability in energy absorption per reaction center, and RC3 represented electron transport and absolute fluorescence. Differences among treatments in RC3 highlight that subtle effects can be detected multivariately even when univariate ANOVA fails. In summer, two components explained 79.9% of the variance, but no significant treatment effects were observed, indicating that seasonal and environmental factors outweighed biofertilizer effects [60].

Radar plots confirmed that seasonality and interannual variability dominated chlorophyll fluorescence patterns, while biofertilizer effects were minor and inconsistent. Parameters such as *Fv*/*Fm* and *φ_Eo_* were tightly clustered, whereas PI _ABS_ and DIo/RC occasionally diverged, especially during extreme climatic years (2020, 2022). This underscores the high resilience of PSII photochemistry, maintaining functional stability under varying nutritional inputs when conditions are favorable or moderately stressful [61].

Overall, while biofertilizers may induce subtle physiological adjustments detectable in multivariate space (e.g., RC3 in spring), the primary determinants of chlorophyll fluorescence dynamics are seasonal leaf development and interannual climatic variability [62].

### 3.6. Integration of Agronomic and Physiological Responses Across Biofertilizer Treatments—Key Treatment Insights

Table 9 summarizes the agronomic and physiological responses of apple trees to biofertilizer treatments over 2019–2022. Overall, treatment effects were selective: certain biofertilizers enhanced growth and yield traits, while physiological variables remained largely stable.

For growth and production, Myc4, iNM6, and iNM12 consistently improved final fruit diameter and number of fruits per tree, with recurrent positive effects (++/+++) across multiple years. T100 and Myc2 showed occasional improvements (+), but less consistently. Allg12, Allg6, and T70 had limited impact, with mostly non-significant responses (ns). Interannual variability and year × treatment interactions explained much of the variation in parameters such as average fruit weight, rather than a consistent treatment effect.

Yield and quality indicators followed similar patterns. Myc4, iNM6, and iNM12 recurrently increased fruit number, yield, and certain quality attributes (Brix, dry matter), whereas firmness and some other quality traits remained mostly unchanged.

Physiological traits—including *C_i_*, WUE, *E*, *g_s_*, *A_n_*, selected vegetation indices, and chlorophyll fluorescence parameters (*Fv*/*Fm*, *φ_Eo_*, *φ_Do_*, PI _ABS_, ABS/RC, TRo/RC, ETo/RC, and DIo/RC)—were largely unaffected across treatments. ANOVA and PCA confirmed that year and season (spring/summer) were the dominant drivers of variability, with treatment and treatment × year effects mostly non-significant. This stability indicates that biofertilizers did not compromise photosynthesis or water-use efficiency, even when growth and yield were influenced.

Importantly, the agronomic gains observed under a 30% reduction in mineral fertilization suggest that biofertilizers—particularly Myc4, iNM6, and iNM12—may enhance nutrient acquisition and utilization efficiency at the root-soil interface. This likely allows for trees to maintain or even increase carbon allocation to reproductive structures (fruit number and quality) without requiring compensatory upregulation of canopy photosynthesis. Such effects are consistent with microbial-mediated nutrient acquisition and source-sink regulation in perennial crops. For instance, arbuscular mycorrhizal fungi can enhance photosynthetic activity while modulating hormone levels such as GA_3_, affecting carbon allocation and stress resilience [63,64]. Considering the diverse microbial consortia in the tested biofertilizers, including AMF and plant growth-promoting bacteria, similar interactions likely contributed to the observed improvements in orchard performance under reduced fertilizer inputs. We emphasize that the links proposed here between microbial treatments, improved nutrient use, and enhanced yield are hypotheses consistent with the observed patterns and current literature but remain correlative in the absence of direct root/soil microbiome, nutrient flux, or hormonal data; targeted follow-up experiments are required to establish causality.

In summary, biofertilizer selection may influence fruit yield and certain quality parameters, while physiological processes largely remained stable [65,66]. Among treatments, Myc4, iNM6, and iNM12 tended to show slightly higher gains, T100 and Myc2 had intermediate effects, and Allg6, Allg12, and T70 showed limited impact. However, strong interannual variability necessitates cautious interpretation. The generally stable values of physiological indices, sometimes accompanied by modest agronomic gains in growth, yield, and quality, were achieved with a 30% reduction in applied fertilizer units. These observations suggest a potential for biofertilizers to support photosynthetic performance under varying environmental conditions [27,67,68], without implying consistent or large enhancements. Overall, these results highlight the possibility of reducing mineral fertilizer use while contributing to orchard sustainability.

**Table 9:** Summary of agronomic and physiological responses of apple trees to biofertilizer treatments (2018–2022).

Study Type	Theme	Parameter	Year/Season	T100	Myc2	Myc4	iNM6	iNM12	Allg12	Allg6	T70
I [13]	Fruit Set	Fruit Set	2019–2022	ns	ns	ns	ns	ns	ns	ns	ns
	Fruit Growth	Final Diameter	2019	ns	+	+	+	+	++	+++	++
			2020	ns	+	+	+	ns	+	ns	ns
			2021	ns	+	ns	ns	+	ns	+	+
			2022	+	ns	+	+	+	ns	ns	ns
	Fruit Production and Yield	Number of Fruits per Tree	2019	+	++	++	+	ns	ns	++	+
			2020	++	+	+	+	+	ns	ns	++
			2021	+	+	+	++	+	+	+	++
			2022	+	+	++	++	+	+	+	++
		Average Fruit Weight	2019	+	+	+	+	++	+	+	++
			2020	+	++	+	++	+	++	+	+
			2021	++	+	++	++	++	++	+	+
			2022	++	+	++	+	+	+	+	+
		Fruit Production	2019	+	+	++	+	+	ns	++	+
			2020	++	+	+	+	+	ns	ns	++
			2021	++	+	++	++	+	+	+	++
			2022	+	+	++	++	+	+	+	++
		BBI	2019–2022	+++	+	+++	+	+	+	+++	+++
	Fruit Quality	Fruit Diameter	2019	ns/+	+	ns/++	ns/++	+	ns/+	+	+
			2020	ns	++	++	++	ns	+	ns	ns
			2021	+	+	ns	ns	+	ns	+	+
			2022	+	ns	+	+	+	ns	ns	ns
		Average Fruit Weight	2019–2022	ns	ns	ns	ns	ns	ns	ns	ns
		Fruit Firmness	2019	ns	ns	ns	ns	ns	ns	ns	++
			2020	ns	ns	ns	ns	ns	ns	ns	ns
			2021	ns	ns	+	ns	ns	ns	ns	ns
			2022	ns	+	ns	ns	ns	ns	ns	ns
		Brix	2019	+	+	+	+	+	+	+	+
			2020	++	+	+	++	+	++	+	+
			2021	++	+	+	++	+	++	+	+
			2022	++	+	+	+	+	+	+	+
		Dry Matter	2019	+	+	+	+	+	+	+	+
			2020	+	++	+	+	+	+	+	+
			2021	+	+	+	+	+	+	+	+
			2022	++	++	+	++	+	+	+	+
		Hue	2019	+	+	+	++	+	++	+	+
			2020	+	+	+	+	+	++	+	+
			2021	++	+	+	+	+	+	+	+
			2022	++	+	+	+	+	++	+	+
	Fruit Size	Fruit Caliber (65–75 mm)	2019	++	+++	++	+	+	+	++	++
			2020	++	++	++	++	+	+	+	++
			2021	++	++	+++	++	++	+	+	++
			2022	++	++	+++	++	++	+	++	++
II	SLA	SLA	2019–2022 S and Su	ns	ns	ns	ns	ns	ns	ns	ns
	CCI	CCI	2018 Au	+	ns	ns	ns	ns	ns	ns	ns
	Gas Exchange Parameters/ Vegetation Indices/ Chlorophyll Fluorescence	*C_i_*, WUE, *E*, g_s_, *A_n_*/NDVI, OSAVI, SIPI, GM2/*Fv*/*Fm*, *φ_Eo_*, *φ_Do_*, PI _ABS_, ABS/RC, TRo/RC, ETo/RC, DIo/RC	2019–2022 S and Su	ns	ns	ns	ns	ns	ns	ns	ns

Au—autumn; S—spring; Su—summer; ns—no significant effect of treatment (*p* ≥ 0.05); +—treatment effect statistically significant (*p* < 0.05). ++/+++—indicate increasingly notable or consistent treatment effects observed across years/seasons, but are not quantitatively defined. These symbols are meant for visual comparison only and do not represent specific *p*-values or effect sizes. Data for physiological parameters were measured in spring and summer, while agronomic parameters were measured annually (2019–2022).

## 4. Materials and Methods

### 4.1. Experimental Site and Meteorological Data

**Figure 8:**
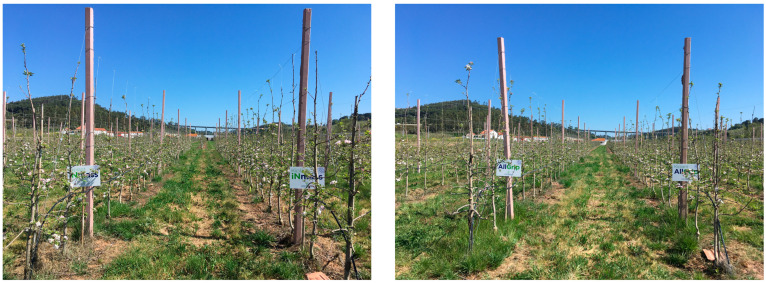
General view of the experimental apple orchard, showing the randomized layout of fertilization treatments.

This study evaluated the multi-year physiological response of ‘Gala’ apple trees to several microbial biofertilizer treatments under commercial orchard conditions in western Portugal. The study was carried out in a drip-irrigated apple orchard (Gala Redlum × M9) established in May 2018 at a density of 3472 trees ha^−1^ (3.20 × 0.90 m spacing) in Alcobaça, central-western Portugal (coordinates of the orchard vertices: 39°33′00″ N, 8°57′40″ W; 39°32′59″ N, 8°57′39″ W; 39°32′59″ N, 8°57′40″ W; 39°32′58″ N, 8°57′39″ W). The region features a transitional Mediterranean climate (C*sb* Köppen–Geiger scale), with mild, wet winters and warm, dry summers [69]. The orchard plot used for the study covered 0.092 ha. Meteorological data (2018–2022) showed strong seasonal patterns, with cool, humid winters; mild springs; and hot, dry summers, occasionally resulting in potential water stress periods (Table 10). Two photos of the orchard (Figure 8) illustrate the experimental layout and different treatment rows. Soil type, texture, and irrigation details are described in Part I of this work [13].

**Table 10:** Seasonal averages (±SD) of meteorological parameters during the 2018–2022 study period.

Season	T_mean_ (°C)	RH_mean_ (%)	RS (MJ m^−2^ d^−1^)	Total Precipitation (mm)	Total ET_o_ (mm)
Winter	11.1 ± 1.2	80.2 ± 5.3	8.7 ± 1.2	247 ± 63	125 ± 21
Spring	14.3 ± 0.4	78.2 ± 5.1	18.1 ± 2.3	215 ± 78	286 ± 27
Summer	20.4 ± 0.7	76.0 ± 4.5	22.8 ± 2.8	26 ± 13	423 ± 47
Autumn	16.2 ± 0.7	77.7 ± 5.0	13.8 ± 2.2	227 ± 59	175 ± 44

T_mean_—seasonal mean air temperature; RH_mean_—seasonal mean relative humidity; RS—seasonal mean solar radiation; ET_o_—seasonal reference evapotranspiration (sum over the season). Values represent averages ± standard deviation over five consecutive years.

### 4.2. Fertilization Plan

Eight fertilization treatments were applied: two mineral controls (T100, 100% conventional dose; T70, 70% conventional dose) and six biofertilizer treatments combining reduced mineral fertilization with Mycoshell^®^, Kiplant iNmass^®^, or Kiplant All-Grip^® ^(Asfertglobal, Santarém, Portugal). [13]. Mycoshell^®^ tablets were applied at planting, while liquid biofertilizers were applied annually around the root zone.

The experiment followed a randomized complete block design with four replications per treatment. Each replication consisted of a row segment with approximately 40 trees planted. Within each treatment row (replicate), ten trees were randomly selected at the beginning of the experiment and permanently tagged to serve as fixed experimental units throughout the study, ensuring uniform exposure and avoiding edge effects. Border trees were excluded. Mineral fertilization was split into two applications per season (T0: spring, T1: summer), with slight adjustments in 2022 to optimize nutrient availability.

Table 11 summarizes the treatments, row lines, and application time. This table was adapted from [13] to maintain clarity while reducing direct text overlap.

**Table 11:** Fertilization treatments applied in the experimental apple orchard, including treatment codes, row lines, application rates, and timing.

Treatment	Line	Description	Application Timing
T100	L7	Control—100% conventional fertilization	According to crop cycle
Myc2	L8	Mycoshell^®^ (2 tablets per tree)	At planting
Myc4	L9	Mycoshell^®^ (4 tablets per tree)	At planting
iNM6	L10	Kiplant iNmass^®^ (6 L ha^−1^ annually)	March annually
iNM12	L11	Kiplant iNmass^®^ (12 L ha^−1^ annually)	March annually
Allg6	L12	Kiplant All-Grip^®^ (6 L ha^−1^ annually)	March annually
Allg12	L13	Kiplant All-Grip^®^ (12 L ha^−1^ annually)	March annually
T70	L14	Control—70% conventional fertilization	According to crop cycle

### 4.3. Physiological Measurements

#### 4.3.1. Measurement of Specific Leaf Area (SLA)

SLA was measured over four consecutive years (2019–2022), with two sampling campaigns conducted each year, one in late spring and another in summer. Leaf measurements were conducted over four years (2019–2022) with two campaigns per year (spring and summer). In 2019, 24 leaf replicates per date were collected (8 treatments × 3 replicates), while in 2020–2022, 40 leaf replicates per date were collected (8 treatments × 5 replicates). Leaves were collected from the middle third of the canopy, selecting well-exposed and healthy leaves free of visible lesions or phytosanitary problems, and were immediately placed in sealed containers to prevent moisture loss during transport.

For each treatment, three Petri dishes were prepared in 2019 and five dishes in 2020–2022, corresponding to the number of leaf replicates collected each year. Each dish consistently contained 12 leaf disks obtained from six leaves across all sampling dates and years. Fresh mass was determined using an analytical balance (Kern ALJ 160-4AM, max 180 g, precision 0.1 mg; Kern & Sohn GmbH, Balingen, Germany). The disks were then oven-dried at 70 °C for 48 h in a static-air oven (WTC Binder 7200, Typ E 115; Tuttlingen, Germany) and weighed immediately after drying using the same balance to determine dry mass.

SLA was calculated following Reich et al. [70] using the expression:

(1) $$SLA(\frac{{cm}^{2}}{g})=\pi \times {\left(\frac{disc\;diameter}{2}\right)}^{2}\times \;number\;of\;discs\;\times \;\frac{1}{Dry\;weight\;discs}$$

#### 4.3.2. Measurement of Chlorophyll Content Index (CCI)

From 2018 to 2020, chlorophyll content was assessed using a SPAD-502 Plus chlorophyll meter (Konica Minolta, Osaka, Japan). This device estimates relative chlorophyll concentration by measuring leaf transmittance at two wavelengths: 650 nm (red), where chlorophyll strongly absorbs light, and 940 nm (near-infrared), which serves as a reference wavelength minimally affected by chlorophyll content. The SPAD-502 Plus provides dimensionless SPAD units proportional to chlorophyll concentration, with a measurement area of 2 × 3 mm, a resolution of ±1 SPAD unit, and high repeatability for in-field applications.

Due to equipment unavailability, no data were collected in 2021. Since 2022, measurements were performed with the MC-100 Chlorophyll Concentration Meter (Apogee Instruments, Logan, UT, USA). This hand-held device determines relative chlorophyll content by measuring the ratio of leaf transmittance at two wavelengths: 653 nm (red) and 931 nm (near-infrared), corresponding to photosynthetically active and reference spectral regions, respectively. The MC-100 utilizes paired detectors and light-emitting diodes to calculate transmittance ratios, internally converted to estimated chlorophyll concentration expressed in µmol m^−2^ of leaf surface.

SPAD values were converted to CCI units according to the regression equation reported by Parry et al. [71], which demonstrated a strong linear relationship (R^2^ > 0.9) between SPAD readings and absolute chlorophyll concentration across several species, including pome fruits:

(2) $$CCI=\frac{SPAD-4.2}{1.5}$$

This conversion was applied to facilitate comparative trend analysis across years and instruments, while acknowledging that absolute chlorophyll values obtained from SPAD and MC-100 sensors may differ.

To account for the influence of the phenological stage, measurement dates were grouped into three seasonal periods: S1 (spring)—including 17 May and 3 June 2019, 4 June 2020, and 27 May 2022—corresponding to the full flowering and early fruit set stages; S2 (summer)—including 12 July, 16 August, and 1 August 2019, 4 August 2020, and 27 July and 8 August 2022—representing the fruit development and ripening phase; and S3 (post-harvest)—including 26 September and 21 November 2018—corresponding to the late season and leaf senescence period. All measurements were taken on fully expanded, healthy, sun-exposed leaves from the mid-portion of current-season shoots. Tree means were used for all statistical analyses.

Each treatment consisted of 8 treatments × 10 trees per measurement date, except on 4 August 2020, when 8 × 6 trees were sampled due to temporary limitations. In 2021, no measurements were collected due to equipment unavailability.

#### 4.3.3. Measurement of Gas Exchange Parameters

Gas exchange was measured on fully expanded, sun-exposed leaves collected from apple trees specifically selected according to the experimental design and subjected to each treatment between 2019 and 2022. For each campaign, the number of leaf replicates per treatment is reported to provide clarity on the sampling effort (e.g., 21 August 2019: 8 treatments × 4 leaves, total 32; 3 June and 4 August 2020: 8 × 5 leaves, 40 per date; 26 May 2021: 8 × 6 leaves, 48 in total; 2 June and 27 July 2022: 8 × 6 leaves, 48 per date).

Gas exchange measurements were performed in the morning (09:30–13:00 h) under clear-sky conditions using a portable infrared gas analyzer (IRGA, LCproT, ADC Bioscientific Ltd., Hoddesdon, UK) coupled to a leaf chamber (6.35 cm^2^). Leaves from the outer canopy were acclimated for 2–3 min prior to recording steady-state values. The CO_2_ concentration was set at ≈400 µmol mol^−1^, incident photosynthetically active radiation (PAR) at 1500 µmol m^−2^ s^−1^, leaf chamber temperature at 25 °C, and airflow through the chamber at 200 µmol s^−1^. This PAR level was selected to ensure light-saturating conditions for all measurements and to standardize gas exchange assessments across treatments, seasons, and years.

For each leaf, the net photosynthetic rate (*A_n_*, µmol CO_2_ m^−2^ s^−1^), stomatal conductance (*g_s_*, mol H_2_O m^−2^ s^−1^), transpiration rate (*E*, mmol H_2_O m^−2^ s^−1^), and intercellular CO_2_ concentration (*C_i_*, µmol mol^−1^) were recorded. Instantaneous water-use efficiency (WUE) was calculated as the ratio of net photosynthesis to transpiration according to:

(3) $$WUE=\frac{An}{E}$$

Due to its integrative nature over the growing season, WUE was analyzed as an annual parameter.

Data for *A_n_*, *E*, and *g_s_* were analyzed seasonally to account for variation between spring and summer, whereas *C_i_* and WUE were analyzed across the full dataset, as no significant seasonal differences were detected for these parameters.

#### 4.3.4. Measurement of Leaf Reflectance Indices

Leaf reflectance indices were measured on fully expanded, sun-exposed leaves collected from apple trees specifically marked according to the experimental design and subjected to each treatment between 2019 and 2022 using a PolyPen RP 410 UVIS spectroradiometer (Photon Systems Instruments, Drásov, Czech Republic), which operates over a spectral range of 380–790 nm. The device includes an integrated xenon light source and measures reflectance via a non-destructive leaf clip. Reflectance spectra were used to calculate 25 vegetation indices (NDVI, SRI, MCARI1, OSAVI, G, MCARI, TCARI, TVI, ZMI, SRPI, NPQI, PRI, NPCI, Ctr1, Ctr2, Lic1, Lic2, SIPI, GM1, GM2, ARI1, ARI2, CRI1, CRI2, and RDVI), whose full names and formulas are provided in Table A5, allowing for clear identification and reproducibility of each index.

Measurements were conducted on one leaf per tree for each treatment, ensuring that each leaf represented a distinct marked tree. For 2019, measurements were performed on 13 June and 14 August; for 2020, on 3 June and 4 August; for 2021, on 26 May and 12 August; and for 2022, on 27 May and 27 July. The PolyPen was calibrated with a Spectralon^®^ white reflectance standard [72] before each session according to the manufacturer’s instructions. Reflectance spectra and calculated indices were recorded in real time, stored in the device’s internal memory, and transferred via USB for further analysis. These indices provided insights into pigment content and the physiological status of apple trees across four growing seasons under different fertilization treatments, enabling temporal comparisons and assessment of treatment effects.

#### 4.3.5. Chlorophyll Fluorescence

Chlorophyll fluorescence was assessed between 2019 and 2022 using two different instruments. In 2019, 2020, and 2021, measurements were performed with a FluorPen FP 110 (PSI, Brno, Czech Republic). In these years, chlorophyll fluorescence was recorded on eight treatments, using 10 replicates in 2019, 5 replicates in 2020, and 6 replicates in 2021, corresponding to 13 June and 14 August 2019, 3 June and 4 August 2020, and 26 May and 12 August 2021, respectively. The FluorPen provided values for the main OJIP-derived parameters, including *Fv*/*Fm*, *φ_Eo_*, *ΦD*_0_, PI _ABS_, ABS/RC, TRo/RC, ETo/RC, and DIo/RC, among others, with leaves dark-adapted for 20 min.

In 2022, chlorophyll fluorescence was measured using a HandyPEA Plant Efficiency Analyzer (Hansatech Instruments Ltd., Norfolk, UK) on 2 June and 27 July, with eight treatments × six replicates (*n* = 48). Leaves were also dark-adapted for 20 min using leaf clips before being exposed to a saturating pulse of 3500 µmol photons m^−2^ s^−1^.

Because FluorPen and HandyPEA differ in excitation pulse, detectors, and algorithms, only *Fv*/*Fm* was compared across all years; all other parameters were analyzed exclusively for 2019–2021.

### 4.4. Statistical Analysis

All statistical analyses were performed using treatment means per tree, with a significance level of α = 0.05, unless otherwise stated. Seasonal data (including SLA) were analyzed separately for spring and summer. Descriptive statistics (mean ± SEM) were computed for all parameters. Assumptions of normality and homoscedasticity were assessed using Shapiro–Wilk and Levene’s tests. Two-way ANOVA was used to test treatment, year, and interaction effects, with Tukey’s HSD or Games–Howell for post hoc comparisons. Effect sizes were expressed as partial eta squared (η^2^p).

PCA was used to explore multivariate patterns among physiological variables and treatments, as several indices showed expected multicollinearity, justifying dimensionality reduction. Vegetation indices and chlorophyll fluorescence parameters were also subjected to PCA, performed separately for spring and summer. Sampling adequacy was verified by KMO and Bartlett tests. Components with eigenvalues > 1 were retained, and Promax rotation was applied. Principal component scores were analyzed by ANOVA with corresponding post hoc tests.

All analyses were conducted in JASP v0.19.1 (University of Amsterdam, The Netherlands). Given the multiple factors and repeated testing across years and seasons, we acknowledge that the risk of Type I error may be inflated. However, most effects were non-significant, and results were interpreted with caution, considering interannual and seasonal variability.

## 5. Conclusions

This multiyear study demonstrated that the physiological performance of ‘Gala’ apple orchards was mainly shaped by interannual climatic variability, with biofertilizer treatments showing minimal and inconsistent effects on leaf morphology, chlorophyll content, reflectance indices, gas exchange, or chlorophyll fluorescence parameters across years and seasons. These findings indicate that the photosynthetic apparatus and canopy function remained largely stable compared with trees fertilized under standard programs (T100), suggesting that microbial inoculants may support, rather than substantially enhance, physiological stability in orchards.

Nevertheless, although the field experiment extended over multiple years, physiological responses were strongly season- and year-dependent, which may limit the direct reproducibility of these physiological patterns across seasons and environmental conditions. This limitation should be considered when extrapolating the present physiological results beyond the specific climatic context of the study period.

Importantly, despite the limited physiological effects, Part I of this study showed that certain biofertilizers—particularly Myc4, iNM6, and iNM12—consistently improved fruit growth, yield, and selected quality traits, even under a 30% reduction in mineral fertilizer application. Part II explains those results and demonstrates that biofertilizers can enhance agronomic performance without altering key physiological processes, supporting a strategy for reducing mineral fertilizer inputs while maintaining productivity.

In summary, while biofertilizers did not significantly modulate the physiological indices measured, their application enabled substantial agronomic gains under reduced nutrient inputs, illustrating their potential to improve orchard sustainability. Future research should integrate physiological, agronomic, and soil-based indicators to identify conditions under which microbial inoculants can maximize crop performance and resilience, particularly under environmental stress.

## Figures and Tables

**Table 1:** CCI (mean ± SEM) for each treatment across seasons. Different letters (bold) indicate significant differences among treatments in autumn 2018 (Tukey’s HSD, *p* < 0.05). No letters are shown for spring and summer because pairwise contrasts were not significant.

Treatment	Spring	Summer	Autumn
T100	28.49 ± 0.67	36.69 ± 1.18	33.09 ± 0.83 **b**
Myc2	31.89 ± 1.18	35.41 ± 0.86	31.44 ± 0.49 **ab**
Myc4	34.16 ± 1.71	35.52 ± 0.81	30.75 ± 0.58 **ab**
iNM6	34.88 ± 1.86	36.03 ± 0.93	31.97 ± 0.99 **ab**
iNM12	32.87 ± 1.28	35.11 ± 0.81	31.60 ± 0.90 **ab**
Allg6	32.14 ± 1.40	35.28 ± 0.82	29.99 ± 0.58 **a**
Allg12	32.84 ± 1.03	36.17 ± 0.84	30.82 ± 0.42 **ab**
T70	32.09 ± 1.02	34.94 ± 0.80	29.90 ± 0.58 **a**

**Table 2:** Intercellular CO_2_ concentration (*C_i_*, µmol mol^−1^, mean ± SEM) across treatments and years.

Treatment	2019	2020	2021	2022
T100	252.25 ± 7.06	261.40 ± 4.39	262.83 ± 13.91	234.00 ± 15.09
Myc2	257.50 ± 9.18	268.30 ± 6.38	259.83 ± 6.30	241.42 ± 12.94
Myc4	263.00 ± 6.16	264.20 ± 6.92	282.33 ± 6.79	256.67 ± 14.68
iNM6	256.25 ± 4.96	268.10 ± 3.90	279.83 ± 14.19	248.58 ± 14.82
iNM12	269.50 ± 4.05	262.30 ± 2.59	255.50 ± 4.00	238.67 ± 14.84
Allg6	262.00 ± 9.60	264.70 ± 6.15	260.00 ± 7.08	232.42 ± 10.83
Allg12	265.25 ± 6.41	258.70 ± 3.90	265.67 ± 7.41	252.83 ± 10.93
T70	267.50 ± 6.55	273.80 ± 6.20	281.67 ± 3.89	234.42 ± 15.14

**Table 3:** Water-use efficiency (WUE, µmol CO2 mmol−1, mean ± SEM) across treatments and years.

Treatment	2019	2020	2021	2022
T100	4.94 ± 0.31	5.12 ± 0.14	4.55 ± 0.33	5.33 ± 0.41
Myc2	4.62 ± 0.46	4.63 ± 0.13	4.35 ± 0.15	5.21 ± 0.37
Myc4	4.60 ± 0.66	4.70 ± 0.12	4.21 ± 0.23	4.77 ± 0.33
iNM6	4.78 ± 0.37	4.89 ± 0.13	3.89 ± 0.40	4.93 ± 0.41
iNM12	4.18 ± 0.59	5.08 ± 0.11	4.42 ± 0.18	5.24 ± 0.33
Allg6	4.26 ± 0.50	4.75 ± 0.22	4.41 ± 0.18	5.37 ± 0.23
Allg12	4.25 ± 0.27	4.96 ± 0.10	4.04 ± 0.25	4.79 ± 0.26
T70	4.03 ± 0.20	4.92 ± 0.21	3.71 ± 0.20	5.17 ± 0.39

**Table 4:** Rotated component loadings from PCA of vegetation indices (spring + summer). Only loadings ≥ 0.4 are shown; strong loadings (≥0.7) are in bold. Promax rotation applied.

Component	Variance Explained (%)	Cumulative (%)	Representative Variables (Loadings ≥ 0.4)
RC1	48.8	48.8	***A_n_***** 1.09, *****g_s_***** 0.83,** *E* 0.69
RC2	46.9	95.7	***C_i_*** ** 0.88, WUE −1.08**

**Table 5:** Rotated component loadings from PCA of vegetation indices (spring). Only loadings ≥ 0.4 are shown; strong loadings (≥0.7) are in bold. Promax rotation applied.

Component	Variance Explained (%)	Cumulative (%)	Representative Indices (Loadings ≥ 0.4)
RC1	55.4	55.4	**ZMI 1.07**, GM2 1.02, TCARI 0.97, Ctr2 −0.96, MCARI −0.96, **TVI −0.92**, **MCARI1 −0.92**, **GM1 0.89**, **NDVI 0.81**, **SRI 0.77**, **Lic1 0.74**, **OSAVI 0.73**, **SIPI 0.72**, CRI1 0.58, RDVI 0.57, CRI2 0.56, NPCI 0.45, ARI1 0.45, ARI2 0.43
RC2	31.6	87.1	**Lic2 −0.89**, **Ctr1 0.89**, **PRI 0.79**, **G 0.72**, ARI2 0.68, ARI1 0.67, NPCI 0.61, CRI2 0.56, SRPI −0.63, CRI1 0.50, SIPI 0.41

**Table 6:** Rotated component loadings from PCA of vegetation indices (summer). Only loadings ≥ 0.4 are shown; strong loadings (≥0.7) are in bold. Promax rotation applied.

Component	Variance Explained (%)	Cumulative (%)	Representative Indices (Loadings ≥ 0.4)
RC1	50.40	50.40	**GM2 1.01**, **Ctr2 −1.01**, **SIPI 0.99**, **NDVI 0.99**, **GM1 0.98**, **SRI 0.98**, **Lic1 0.98**, **CRI1 0.93**, **OSAVI 0.91**, **TCARI 0.97**, **CRI2 0.87**, **PRI 0.70**, RDVI 0.64, G 0.57
RC2	25.00	75.40	**ARI2 1.03**, **ARI1 1.02**, **Ctr1 1.00**, **Lic2 −0.97**, **NPCI 0.82**, CRI2 0.42, **SRPI −0.81**, NPQI −0.65
RC3	16.90	92.40	**TVI 0.99**, **MCARI1 0.98**, **MCARI 0.96**, TCARI −0.42, ZMI −0.41, RDVI 0.58

## Data Availability

The original contributions presented in this study are included in the article. Further inquiries can be directed to the corresponding authors.

## Abbreviations

The following abbreviations are used in this manuscript:

ABS/RC	Absorption flux per reaction center
*A_n_*	Net photosynthetic rate
ARI1	Anthocyanin Reflectance Index 1
ARI2	Anthocyanin Reflectance Index 2
BBI	Biennial Bearing Index
*C_i_*	Intercellular CO_2_ concentration
Ctr1	Carter Index 1
Ctr2	Carter Index 2
CRI1	Carotenoid Reflectance Index 1
CRI2	Carotenoid Reflectance Index 2
DIo/RC	Dissipated energy flux per reaction center
*E*	Transpiration rate
ETo/RC	Electron transport flux per reaction center
Fv/Fm	Maximum quantum yield of PSII
G	Greenness Index
GM1	Gitelson and Merzlyak Index 1
GM2	Gitelson and Merzlyak Index 2
*gs*	Stomatal conductance
KMO	Kaiser–Meyer–Olkin
Lic1	Leaf Chlorophyll Index 1
Lic2	Leaf Chlorophyll Index 2
MCARI	Modified Chlorophyll Absorption in Reflectance Index
MCARI1	Modified Chlorophyll Absorption in Reflectance Index 1
NDVI	Normalized Difference Vegetation Index
NPCI	Normalized Pigment Chlorophyll Index
NPQI	Normalized Phaeophytinization Index
OSAVI	Optimized Soil-Adjusted Vegetation Index
PAR	Photosynthetically active radiation
PCA	Principal component analysis
PI _ABS_	Performance index on absorption basis
*φ_Do_* (phi_Do)	Quantum yield of energy dissipation
*φ_Eo_* (phi_Eo)	Quantum yield for electron transport
PRI	Photochemical Reflectance Index
RDVI	Renormalized Difference Vegetation Index
SD	Standard deviation
SEM	Standard error of the mean
SIPI	Structure-Insensitive Pigment Index
RS	Solar radiation
SRI	Simple Ratio Index
SRPI	Simple Ratio Pigment Index
TCARI	Transformed Chlorophyll Absorption in Reflectance Index
T_max_	Maximum daily air temperature (°C)
T_med_	Mean daily air temperature (°C)
T_min_	Minimum daily air temperature (°C)
TRo/RC	Trapped energy flux per reaction center
TVI	Triangular Vegetation Index
ZMI	Zarco-Tejada & Miller Index
WUE	Water-use efficiency

## Appendix A

### Appendix A.1. Two-Way ANOVA of Specific Leaf Area (SLA) Across Treatments and Years

**Table A1:** Two-way ANOVA of SLA for spring and summer measurements showing the effects of treatment, year, and their interaction.

Season	Factor	F	*p*-Value
Spring	Treatment	1.30	0.256
	Year	313.61	<0.001
	Treatment × Year	2.49	0.001
Summer	Treatment	3.72	0.001
	Year	572.45	<0.001
	Treatment × Year	1.87	0.019

### Appendix A.2. Summary of ANOVA for Gas Exchange and Physiological Variables

**Table A2:** Summary of ANOVA results for all physiological variables (*C_i_*, WUE, *E*, *g_s_*, and *A_n_*) across treatments, years, and their interaction.

Variable	Season	Effect	F	*p*	η^2^p	Significant?
*C_i_*	Spring	Treatment	0.61	0.75	0.02	
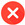
	Spring	Year	10.09	<0.001	0.12	
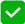
	Spring	Trt × Year	0.39	0.99	0.04	
WUE	Summer	Treatment	0.90	0.51	0.03	
	Summer	Year	12.81	<0.001	0.15	
	Summer	Trt × Year	0.48	0.97	0.04	
*E*	Spring	Treatment	0.67	0.70	0.04	
	Spring	Year	42.55	<0.001	0.43	
	Spring	Trt × Year	0.77	0.70	0.09	
	Summer	Treatment	1.44	0.20	0.10	
	Summer	Year	4.25	0.02	0.08	
	Summer	Trt × Year	1.14	0.33	0.14	
*g_s_*	Spring	Treatment	1.32	0.25	0.08	
	Spring	Year	39.83	<0.001	0.42	
	Spring	Trt × Year	0.76	0.71	0.09	
	Summer	Treatment	1.17	0.33	0.08	
	Summer	Year	9.24	<0.001	0.16	
	Summer	Trt × Year	0.98	0.48	0.13	
*A_n_*	Spring	Treatment	0.85	0.55	0.05	
	Spring	Year	25.34	<0.001	0.31	
	Spring	Trt × Year	1.41	0.16	0.15	
	Summer	Treatment	0.56	0.79	0.04	
	Summer	Year	22.51	<0.001	0.32	
	Summer	Trt × Year	1.31	0.22	0.16	

### Appendix A.3. ANOVA and Kruskal–Wallis Results for Vegetation Indices

**Table A3:** ANOVA summary for vegetation indices across biofertilizer treatments (2019–2022) during spring and summer seasons.

Vegetation Index	ANOVA F (Spring)	ANOVA *p*-Value (Spring)	ANOVA F (Summer)	ANOVA *p*-Value (Summer)
NDVI	0.023	1.000	0.051	1.000
SRI	0.024	1.000	0.029	1.000
MCARI1	0.181	0.987	0.438	0.869
OSAVI	0.036	1.000	0.045	1.000
G	0.701	0.671	0.212	0.979
MCARI	0.661	0.702	0.411	0.886
TCARI	0.251	0.978	0.178	0.988
TVI	0.183	0.986	0.416	0.883
ZMI	0.372	0.910	0.370	0.911
SPRI	0.023	1.000	0.009	1.000
NPQI	0.129	0.995	0.230	0.974
PRI	0.232	0.973	0.070	0.999
NPCI	0.018	1.000	0.018	1.000
CTR1	0.087	0.999	0.113	0.997
CTR2	0.131	0.995	0.051	1.000
LIC1	0.027	1.000	0.013	1.000
LIC2	0.101	0.998	0.048	1.000
SIPI	0.011	1.000	0.030	1.000
GM1	0.111	0.997	0.096	0.998
GM2	0.196	0.983	0.104	0.998
ARI1	0.011	1.000	0.047	1.000
ARI2	0.013	1.000	0.046	1.000
CRI1	0.024	1.000	0.099	0.998
CRI2	0.010	1.000	0.078	0.999
RDVI	0.084	0.999	0.202	0.982

### Appendix A.4. Seasonal Means of Chlorophyll Fluorescence and Related Parameters

**Table A4:** Seasonal means (±SE) of chlorophyll fluorescence and related parameters across years.

Parameter	Season	2019	2020	2021	2022
*Fv*/*Fm*	Spring	0.80 ± 0.02	0.82 ± 0.02	0.79 ± 0.03	0.78 ± 0.03
*Fv*/*Fm*	Summer	0.79 ± 0.03	0.81 ± 0.02	0.83 ± 0.02	0.80 ± 0.02
*φ_Eo_*	Spring	0.48 ± 0.05	0.49 ± 0.06	0.50 ± 0.03	–
*φ_Eo_*	Summer	0.48 ± 0.05	0.43 ± 0.04	0.51 ± 0.04	–
*φ_Do_*	Spring	0.20 ± 0.02	0.18 ± 0.02	0.19 ± 0.02	–
*φ_Do_*	Summer	0.21 ± 0.03	0.19 ± 0.02	0.16 ± 0.02	–
PI _ABS_	Spring	4.05 ± 1.12	4.21 ± 1.34	5.51 ± 1.76	–
PI _ABS_	Summer	4.85 ± 1.97	4.55 ± 2.11	6.63 ± 2.21	–
ABS/RC	Spring	0.96 ± 0.04	0.97 ± 0.04	1.15 ± 0.05	–
ABS/RC	Summer	1.02 ± 0.04	1.10 ± 0.04	0.99 ± 0.05	–
TRo/RC	Spring	0.99 ± 0.04	0.97 ± 0.04	1.11 ± 0.05	–
TRo/RC	Summer	0.98 ± 0.03	1.04 ± 0.04	1.10 ± 0.04	–
ETo/RC	Spring	0.61 ± 0.05	0.60 ± 0.08	0.68 ± 0.05	–
ETo/RC	Summer	0.67 ± 0.06	0.66 ± 0.08	0.68 ± 0.05	–
DIo/RC	Spring	0.27 ± 0.05	0.26 ± 0.05	0.34 ± 0.10	–
DIo/RC	Summer	0.29 ± 0.06	0.28 ± 0.06	0.22 ± 0.05	–

### Appendix A.5. Vegetation Indices Used in This Study

**Table A5:** List of vegetation indices calculated from PolyPen RP 400 leaf reflectance measurements (2019–2022).

Index	Equation	Reference
NDVI	(R_NIR − R_RED)/(R_NIR + R_RED)	[73]
SR	R_NIR/R_RED	[74]
MCARI1	1.2 × [2.5 × (R_790 − R_670) − 1.3 × (R_790 − R_550)]	[75]
OSAVI	(1 + 0.16) × (R_790 − R_670)/(R_790 − R_670 + 0.16)	[76]
G	R_554/R_677	[77]
MCARI	[(R_700 − R_670) − 0.2 × (R_700 − R_550)] × (R_700/R_670)	[78]
TCARI	3 × [(R_700 − R_670) − 0.2 × (R_700 − R_550) × (R_700/R_670)]	[79]
TVI	0.5 × [120 × (R_750 − R_550) − 200 × (R_670 − R_550)]	[80]
ZMI	R_750/R_710	[81]
SPRI	R_430/R_680	[82]
NPQI	(R_415 − R_435)/(R_415 + R_435)	[83]
PRI	(R_531 − R_570)/(R_531 + R_570)	[84]
NPCI	(R_680 − R_430)/(R_680 + R_430)	[85]
Ctr1	R_695/R_420	[86]
Ctr2	R_695/R_760	[86]
Lic1	(R_550 − R_670)/(R_550 + R_670)	[87]
Lic2	(R_560 − R_710)/(R_560 + R_710)	[87]
SIPI	(R_790 − R_450)/(R_790 − R_650)	[88]
GM1	R_750/R_550	[89]
GM2	R_750/R_700	[89]
ARI1	1/R_550 − 1/R_700	[90]
ARI2	R_790 × (1/R_550 − 1/R_700)	[90]
CRI1	1/R_510 − 1/R_550	[91]
CRI2	1/R_510 − 1/R_700	[91]
RDVI	(R_780 − R_670)/sqrt (R_780 + R_670)	[92]
